# The differential response to Fgf signalling in cells internalized at different times influences lineage segregation in preimplantation mouse embryos

**DOI:** 10.1098/rsob.130104

**Published:** 2013-11

**Authors:** Samantha A. Morris, Sarah J. L. Graham, Agnieszka Jedrusik, Magdalena Zernicka-Goetz

**Affiliations:** 1 Wellcome Trust/Cancer Research UK Gurdon Institute, University of Cambridge, Tennis Court Road, Cambridge CB2 1QR, UK; 2 Department of Physiology, Development and Neuroscience, University of Cambridge, Downing Street, Cambridge CB2 3DY, UK

**Keywords:** mouse embryo, cell lineage, heterogeneity, Fgf signalling, bias

## Abstract

Lineage specification in the preimplantation mouse embryo is a regulative process. Thus, it has been difficult to ascertain whether segregation of the inner-cell-mass (ICM) into precursors of the pluripotent epiblast (EPI) and the differentiating primitive endoderm (PE) is random or influenced by developmental history. Here, our results lead to a unifying model for cell fate specification in which the time of internalization and the relative contribution of ICM cells generated by two waves of asymmetric divisions influence cell fate. We show that cells generated in the second wave express higher levels of Fgfr2 than those generated in the first, leading to ICM cells with varying Fgfr2 expression. To test whether such heterogeneity is enough to bias cell fate, we upregulate Fgfr2 and show it directs cells towards PE. Our results suggest that the strength of this bias is influenced by the number of cells generated in the first wave and, mostly likely, by the level of Fgf signalling in the ICM. Differences in the developmental potential of eight-cell- and 16-cell-stage outside blastomeres placed in the inside of chimaeric embryos further support this conclusion. These results unite previous findings demonstrating the importance of developmental history and Fgf signalling in determining cell fate.

## 2. Introduction

**Figure 1.**
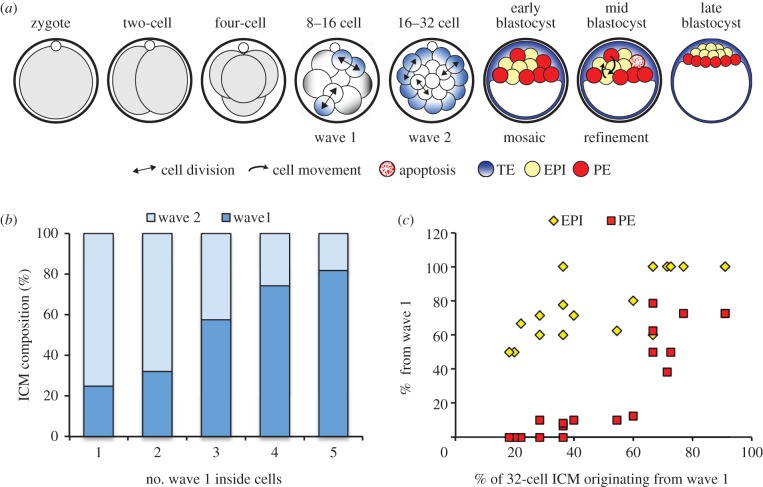
Lineage specification in the preimplantation mouse embryo. (*a*) Preimplantation mouse embryo development. Asymmetric divisions at the 8–16 and 16–32 cell transitions generate inside and outside cells. Outside cells differentiate into TE, whereas inside cells form the pluripotent ICM. PE and EPI precursors are initially distributed in the ICM in a mosaic pattern before being sorted into position by a combination of cell movement and apoptosis. (*b*) Graph of the number of wave 1 inside cells generated at the 8–16 cell stage transition against the composition of the ICM at E4.5 (*n* = 19, data from [3]). (*c*) Graph showing the percentage of the 32-cell stage ICM originating from the first wave of asymmetric cell divisions against the contribution of wave 1-generated cells to each lineage at E4.5 (*n* = 19, data from [3]).

The mammalian blastocyst prior to implantation comprises three distinct lineages—the trophectoderm (TE) and primitive endoderm (PE), which form mainly extra-embryonic structures, such as the placenta and the yolk sac, and the pluripotent epiblast (EPI), which gives rise to the embryo proper. The correct specification of these lineages is critical for all subsequent development and is initiated at the 8–16, 16–32 and 32–64 cell transitions when three waves of asymmetric cell divisions direct cells to the inside of the embryo [1–4]. Cells on the outside of the embryo will progressively differentiate into TE, while cells on the inside of the embryo form the pluripotent inner-cell-mass (ICM). The ICM is further segregated into the PE and EPI lineages as the blastocyst matures so that by embryonic day 4.5 (E4.5) cells on the surface of the ICM, adjacent to the blastocyst cavity, have differentiated into the PE, and deeper ICM cells form the pluripotent EPI (figure 1*a*).

Owing to the positional differences between the PE and EPI at E4.5, it was initially postulated that these lineages are specified owing to their position alone, with a potential signal from the blastocyst cavity inducing PE differentiation in surface cells [5]. It was then discovered that cells of the early (E3.5) ICM express the respective PE and EPI markers, Gata6 and Nanog, in a mosaic ‘salt and pepper’ distribution, independent of cell position [6]. This was in agreement with lineage-tracing studies that showed that whereas the majority of surface ICM cells contribute to extra-embryonic lineages, some contribute to EPI or are bipotent [7]. These precursor cells are then sorted into the correct position by a combination of active actin-dependent cell movements and apoptosis of incorrectly positioned cells [3,8,9]. The mechanism governing ICM cell fate specification is therefore clearly not solely dependent on cell position, but whether the initial restriction of Gata6 and Nanog expression to certain cells is random or related to developmental history of cells has remained unknown.

Two independent studies attempted to answer this question using different methodologies and arrived at different conclusions. Our own study [3] used non-invasive individual computational cell lineage tracing to follow the development of all cells in the embryo for 2.5 days continuously from the eight-cell stage to the E4.5 blastocyst. We found that the fate of ICM cells was influenced by the time at which they were internalized. Those cells generated by the first wave of asymmetric divisions, at the 8–16 cell transition, were significantly biased to give rise to EPI rather than PE, whereas those generated by the second wave, at the 16–32 cell transition, were biased in a reciprocal manner—towards forming PE rather than EPI. The minor third wave of asymmetric divisions solely contributed to PE. In a parallel study, Yamanaka *et al*. [10] injected single blastomeres at the eight-cell stage with a fluorescent marker, monitored whether they gave rise to the first or second wave inside cells, and then, following transfer to pseudo-pregnant females, assessed lineage contribution of the injected cells to tissues derived from the EPI and PE at E5.5. This study reported no link between division history and ICM cell fate. The reasons behind the discrepancies between these two studies have been discussed [11,12]. However, no clear mechanism of ICM cell fate specification that explains both sets of results has been agreed upon.

Here, we wished to test the hypothesis that the developmental history of cells, specifically the time of cell internalization, influences ICM cell fate, and to attempt to explain how such biases might arise. To this end, we considered the involvement of Fgf signalling because its importance for PE formation has been previously demonstrated [10,13]. We find that ICM cells internalized later in development, by the second wave of asymmetric divisions, express higher levels of Fgfr2 than those internalized earlier, by the first wave. We also demonstrate that this heterogeneity of Fgfr2 expression within the ICM provides a mechanism by which cells internalized in the second wave of asymmetric cell divisions can be directed towards the PE lineage in response to Fgf signals. These new results lead us to propose a model for ICM lineage specification in which both developmental history and the specific ICM composition of the embryo can influence cell fate.

## 3. Results

### 3.1. Lineage contribution depends on proportion of the inner-cell-mass derived from each division wave

In agreement with the regulative nature of mammalian embryo development, the number of ICM cells generated by specific waves of asymmetric cell divisions can vary [3,4,14,15]. ICM occupancy following the first wave directly impacts on the number of second-wave asymmetric divisions, such that in the case where a high number of cells have divided asymmetrically in the first wave, there are few second-wave asymmetric divisions and vice versa. We first wished to determine whether the absolute numbers of inside cells generated in the first two waves of cell internalization would affect lineage contributions in the blastocyst, and if so, in what way. To this end, we re-analysed the datasets from the time-lapse study that followed all inside cells from the time of their generation until their fate specification was established at E4.5 [3]. This revealed that the archetypal 32-cell stage embryo accommodates an ICM of 11 cells, which typically consists of a roughly equal balance of cells derived from the first and second waves of internalization. In this most representative group of embryos, an average of two to three cells (2.84) at the eight-cell stage divide asymmetrically, later representing around six cells in the ICM of the early blastocyst [3] (figure 1*b*). In these cases, there is a very significant bias for first-wave cells to contribute to EPI and second-wave cells to PE. We identified two additional groups of embryos: in embryos with fewer wave 1-generated inside cells, and therefore an ICM predominantly made up of cells originating from wave 2, the PE is composed almost exclusively of cells from wave 2 and the EPI is derived from a mixture of the available population, that is wave 1 and wave 2 cells (figure 1*c*). Thus, these embryos also show bias in lineage allocation. We found that only in embryos with as many as four to five cells at the eight-cell stage dividing asymmetrically is the contribution of the first-wave-generated ICM cells to the PE lineage substantial (figure 1*c*). But even in these embryos, which comprise 25% of all embryos, there is a clear bias because the EPI is almost completely derived from wave 1 cells. The differences in lineage contribution between these groups of embryos suggest that the strength of the ICM cell fate bias depends upon the number of cells generated by the first wave of asymmetric divisions and, correspondingly, the proportion of the ICM derived from each wave.

### 3.2. Differential expression of Fgfr2 in inner-cell-mass cells internalized at different times

**Figure 2.**
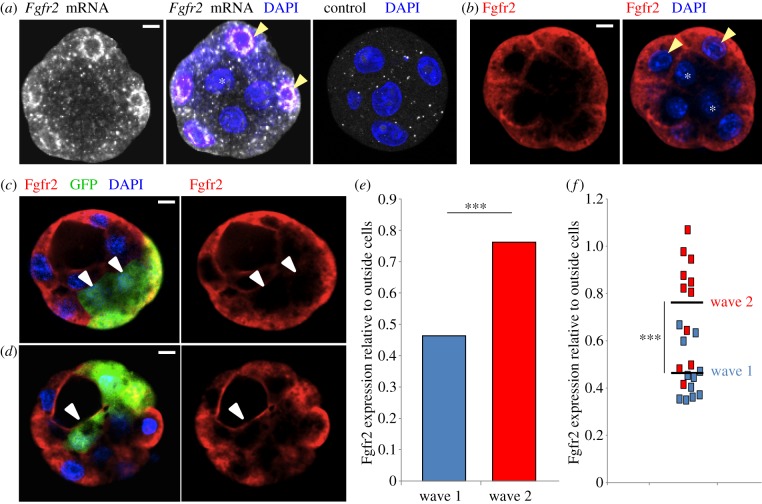
Differential expression of Fgfr2 in ICM cells generated by different waves of asymmetric divisions. (*a*) Fluorescent *in situ* hybridization showing *Fgfr2* mRNA expression in outside cells at the 16-cell stage (*n* = 6, yellow arrow indicates outside cell, asterisk indicates inside cell). (*b*) Immunofluorescence showing Fgfr2 protein expression in outside cells at the 16-cell stage (*n* = 9, yellow arrow indicates outside cell, asterisk indicates inside cell). (*c*,*d*) Fgfr2 expression shown by immunostaining in wave 1 and wave 2 inside cells. Inside cells generated in the first wave of asymmetric cell divisions (*c*, white arrows) express less Fgfr2, than those generated in the second wave (*d*, white arrow). Cells that have been monitored for division history are marked by GFP expression. (*e*,*f*) Quantification of Fgfr2 immunostaining intensity in wave 1- and wave 2-derived inside cells relative to outside cells in the same optical plane (*n* = 22 inside cells and 48 outside cells from 17 embryos, ***p < 0.001). (*e*) Average intensity of Fgfr2-staining. (*f*) Distribution of Fgfr2-staining intensities from (*e*). Scale bars, 10 μm. See also the electronic supplementary material, figure S1.

The above results suggest that (i) a cell fate bias in the preimplantation embryo depends on developmental history of ICM cells and that (ii) this may be masked in embryos with a high proportion of wave 1 inside cells, owing to the regulative nature of development. If this is indeed the case, we argued that there must be some fundamental difference between wave 1 and wave 2 inside cells that creates heterogeneity within the ICM. Several studies have analysed gene expression in the precursors of the PE and EPI at E3.5, and found reciprocal expression of Fgf4 in cells expressing Nanog (EPI precursors) and Fgfr2 in those expressing Gata6 (PE precursors) [16–18]. However, whether the expression of these Fgf signalling components relates to developmental cell history has remained unknown. To determine this, we first analysed the spatial and temporal expression pattern of mRNA and protein of the Fgf signalling pathway receptor expressed in the early mouse embryo, Fgfr2. To determine the expression of Fgfr2 immediately after the first wave of asymmetric divisions, we fixed embryos at the 16-cell stage and processed them either through fluorescent *in situ* hybridization (FISH) to reveal mRNA, or immunostaining to reveal protein. We found higher expression of both *Fgfr2* mRNA and Fgfr2 protein in outside cells than inside cells at the 16-cell stage (figure 2*a*,*b*).

This differential expression of Fgfr2 immediately following the first wave of asymmetric cell divisions suggests that wave 2 inside cells may inherit an increased amount of Fgfr2, as they are the progeny of 16-cell-stage outside cells that have high Fgfr2 expression. To test this hypothesis, we injected individual blastomeres of eight-cell-stage embryos with *GFP* mRNA so that we could monitor asymmetric cell divisions and determine whether labelled inside cells originated from wave 1 or 2 (figure 2*c*,*d*). The embryos were then fixed at the early blastocyst stage to analyse expression of Fgfr2 by immunostaining (figure 2*c*,*d*; electronic supplementary material, figure S1). This revealed that wave 2 inside cells express on average significantly more Fgfr2 than wave 1 inside cells (figure 2*e*,*f*; wave 1: 0.47 relative to outside cells; wave 2: 0.76 relative to outside cells; *p* < 0.001). Both wave 1 and wave 2 inside cells show a range of Fgfr2-staining intensities, with some wave 2-derived inside cells expressing Fgfr2 at a level comparable with outside cells (figure 2*f*). This differential expression of Fgfr2 between ICM cells originating from these two different waves of asymmetric cell divisions indicates that Fgfr2 expression could be involved in regulating their fate through their response to Fgf signalling.

### 3.3. Overexpression of Fgfr2 drives cells towards a primitive endoderm fate

**Figure 3.**
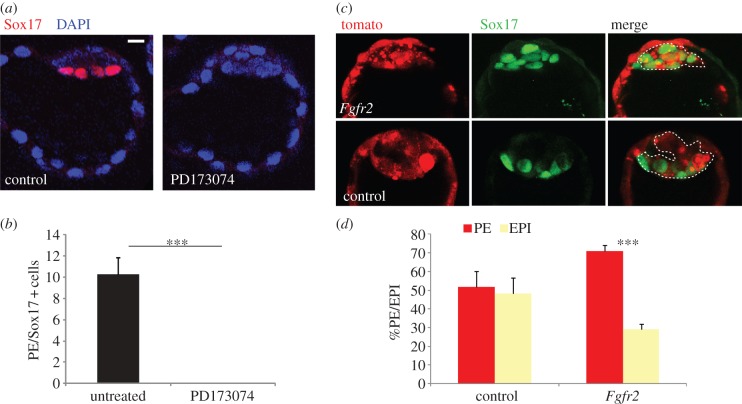
Fgfr2 expression biases cells towards a PE fate. (*a*) Fgfr2 inhibition with the small molecule inhibitor, PD173074 from the eight-cell stage. Left panel: immunostaining of Sox17 in control untreated E4.5 embryo showing defined PE layer. Right panel: absence of Sox17-positive cells in PD173074-treated embryo. (*b*) Quantification is presented (*n* = 12, ***p < 0.001). (*c*) Example of Fgfr2 overexpression in an E4.5 blastocyst following injection of mRNA to one blastomere at the two-cell stage. All *Fgfr2* mRNA-overexpressing ICM cells express Sox17, regardless of their position. In control embryos, only cells on the surface of the ICM express Sox17. Injected cells are marked by Tomato expression and those in the ICM are outlined by a white dashed line. (*d*) Quantification demonstrates Fgfr2-overexpressing cells are driven to PE fate (*n* = 17, ***p < 0.001). Scale bars, 10 μm. See also the electronic supplementary material, figure S2.

To address the hypothesis that the heterogeneity in Fgfr2 expression within the ICM could be enough to influence the fate of individual cells, we first wished to confirm that signalling through Fgfr2 is important for PE formation, as previously reported [10]. To this end, we cultured eight-cell embryos in the presence of the specific Fgfr2 inhibitor PD173074 [19] until the late blastocyst stage (E4.5). Analysis of PD173074-treated embryos by immunofluorescence for Sox17 expression showed a complete absence of PE (figure 3*b*; *p* < 0.001) compared with control embryos, indicating that signalling through Fgfr2 is essential for PE differentiation. To determine whether increased expression of Fgfr2 would be enough to direct cells towards a PE fate, we overexpressed Fgfr2 in part of the embryo and followed cell fate. To do this, we injected one blastomere of the late two-cell-stage embryo with *Fgfr2* mRNA, along with *GFP* or *Tomato* mRNA as a lineage tracer and cultured the embryos to the late blastocyst stage (E4.5; see electronic supplementary material, figure S2). We found that while control-injected cells contributed equally to EPI and PE lineages, Fgfr2-overexpressing ICM cells were directed towards a PE (Sox17-positive) cell fate (figure 3*d*; 71% of injected cells, *p* < 0.001). These results indicate that higher levels of Fgfr2 expression are enough to bias ICM cells to form PE and provide a potential mechanism by which wave 2 inside cells can be directed towards the PE lineage.

### 3.4. Time spent outside influences the fate of internalized cells

**Figure 4.**
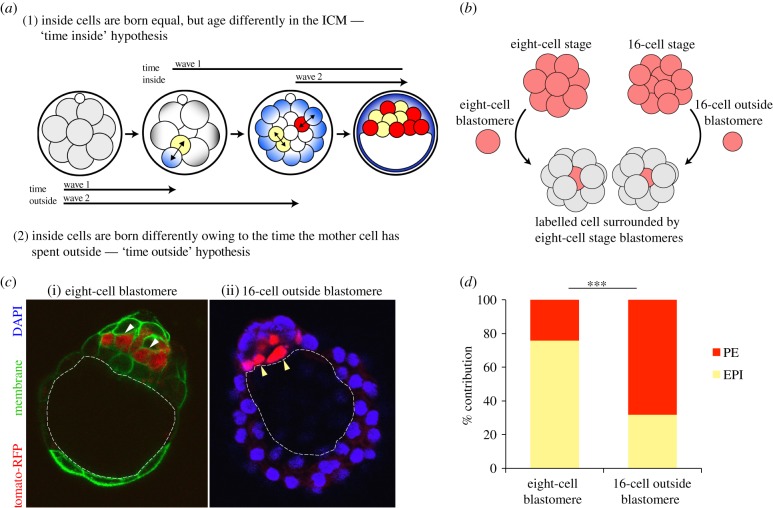
Time outside affects cell fate. (*a*) Two hypotheses for differential lineage bias in ICM cells internalized at different times. (1) In the ‘time-inside’ hypothesis, cells are the same at the time of internalization, but increased time on the inside of the embryo influences cell fate such that cells that have been inside longer are more pluripotent. (2) In the ‘time-outside’ hypothesis, cells are different at the time of internalization owing to the time the mother cell has spent on the outside of the embryo such that when the mother cell stays longer outside, it produces daughter cells that are less pluripotent. (*b*) Creation of chimaeras containing a fluorescently labelled eight-cell blastomere or 16-cell outside blastomere in the centre of the embryo, surrounded by host eight-cell blastomeres. (*c*) Chimaeric embryos at E4.5 showing the progeny of either (i) a single eight-cell stage blastomere or (ii) a 16-cell outside blastomere positioned on the inside of the embryo. The blastocyst cavity is outlined. White arrows indicate deep ICM cells (EPI), yellow arrows indicate surface ICM cells (PE). (*d*) ICM lineage contribution from the labelled cells. Those from eight-cell-stage blastomeres contribute predominantly to EPI (76% EPI, *n* = 99 cells), whereas those from 16-cell-stage outside cells contribute predominantly to PE (68% PE, *n* = 47 cells). ***p < 0.001. Lineage contribution was confirmed by immunostaining (see the electronic supplementary material, figure S3).

The results we present here support the concept that inside cells generated by different waves of asymmetric cell divisions are fundamentally different and suggest that those internalized later are biased towards a PE cell fate by their increased responsiveness to Fgf signalling. This could be a result of the amount of time blastomeres spend on the outside of the embryo influencing the cell fate of their inside progeny (‘time-outside’ hypothesis; figure 4*a*), as is suggested by our Fgfr2 results. Alternatively, this could be owing to wave 1 inside cells developing increased pluripotency owing to their increased time spent in the inside of the embryo (‘time-inside’ hypothesis; figure 4*a*). To address these hypotheses, we generated chimaeric embryos in which either eight-cell stage blastomeres (‘younger’ cells, i.e. spent shorter time outside) or 16-cell stage outside blastomeres (‘older’ cells, i.e. spent longer time outside) were placed inside the embryo, surrounded by host eight-cell stage cells (figure 4*b*). These chimaeras were then cultured to E4.5 and the contribution of the labelled ‘young’ or ‘old’ cells to each ICM lineage assessed by immunofluorescence (figure 4*c*,*d*; electronic supplementary material, figure S3). We found that the ‘older’ 16-cell-stage blastomeres, which had spent more time on the outside of the embryo, were biased to contribute to PE (figure 4*d*; 68% PE). Conversely, the ‘younger’ eight-cell stage blastomeres were biased to contribute to EPI (figure 4*d*; 24% PE). These results suggest that as cells on the outside of the embryo mature, it shifts the cell fate bias of any inside progeny of asymmetric divisions away from the more pluripotent EPI and towards the more differentiated PE lineage. Although these are manipulated embryos in which it is difficult to control for cell size and/or developmental stage, these results are in agreement with our non-invasive lineage tracing of intact embryos where the earliest inside cells were biased towards EPI, whereas the later internalized cells were biased towards PE [3].

## 4. Discussion

How ICM cells are specified into PE and EPI precursors is an unresolved and important question in understanding cell fate determination in the early mouse embryo. Here, we attempt to resolve discrepancies between seemingly contradictory studies and suggest a model for ICM cell fate specification that combines an influence of developmental cell history with the regulative nature of early mouse development. By tracing the fate of ICM cells internalized at different times until the late blastocyst stage [3], we found that lineage specification was biased according to the wave of asymmetric cell divisions inside cells were derived from. This was in contrast with a parallel study that found no such bias [10]. Attempts have been made to explain why such different results were obtained [11,12]. There are two major differences between the two studies: the time at which cell fate was assessed and the average proportion of the ICM generated by the first and second waves of asymmetric divisions. Here, we first analysed a potential link between the number of inside cells generated by each wave and the strength of the lineage bias. The most typical 32-cell-stage embryo has an ICM comprising, on average, an equal balance of cells derived from wave 1 and wave 2. These embryos show a clear bias of wave 1-derived cells to contribute to EPI and wave 2-derived cells to contribute to PE. However, our analyses identify two further, less typical groups of embryos—those with an ICM predominantly composed of cells originating from wave 1 and those with an ICM mainly composed of cells originating from wave 2 (figure 1*c*). We find that when the ICM is mostly derived from wave 1, the wave 2 inside cells are biased to contribute to PE, but as there are few wave 2 cells, wave 1 cells also form PE as well as all of the EPI. In embryos where the ICM is mostly derived from wave 2, the PE is almost exclusively made from wave 2 cells, whereas the EPI is derived from a mixture of cells from both waves. In the study by Yamanaka *et al*. [10], the first wave was found to generate unusually many (4.8) inside cells, which, using our dataset as a reference, would imply that the ICM would be roughly 80% derived from wave 1 cells (figure 1*b*). In these circumstances, first-wave cells contributed to both PE and EPI, in accordance with our dataset. The number of second-wave cells traced in the Yamanaka *et al*. [10] study was very low; however, there does seem to be a bias towards contribution to PE rather than EPI. Thus, the analyses we present here indicate that these two datasets are compatible when ICM composition is taken into account. Why there are differences in the number of wave 1 and 2 cells between the two studies is not clear, but is likely to represent mouse strain- or experimental method-specific effects [11].

Our data suggest that ICM cells derived from the first wave of asymmetric divisions are more likely to form EPI, whereas those derived from the second wave are more likely to form PE. Here, we show for the first time that these differences relate to and are affected by the differential expression of a factor involved in PE specification between ICM cells internalized at different times. Following the first wave of asymmetric cell divisions, we find that Fgfr2 is expressed substantially more in outside cells compared with inside cells at both the mRNA and protein level (figure 2*a*,*b*). This early differential expression means that second wave inside cells inherit Fgfr2 from their outside progenitors, resulting in a heterogeneous ICM comprising wave 1-generated cells with low Fgfr2 expression and wave 2-generated cells with high Fgfr2 expression (figure 2*c–f*). The importance of Fgf signalling in PE formation is well established [10,13,17,20], but here we show for the first time that a higher level of Fgfr2 expression is sufficient to direct cells towards the PE lineage (figure 3*d*). This differential expression of Fgfr2 between wave 1- and 2-derived ICM cells would explain why wave 2 inside cells are more susceptible to Fgf signalling, and therefore biased towards a PE fate. The Fgf4 signal important for initiating PE development has been shown to be produced by Nanog-expressing cells in the early ICM [13,17,21], and our mRNA deep sequencing analyses at the 16-cell stage demonstrate that *Fgf4* mRNA is expressed 100-fold more in inside cells following the first wave of asymmetric divisions (M. Zernicka-Goetz 2013, personal communication). This suggests that wave 1-derived inside cells are the source of Fgf4 signalling in the ICM. Our conclusion that wave 2 inside cells are biased towards a PE fate owing to inherent differences between the ‘parents’ of wave 1 and 2 inside cells (eight-cell blastomeres and 16-cell outside blastomeres, respectively) is further supported by the finding that these two ‘outside’ cell types show different ICM lineage bias when positioned on the inside of the embryo (figure 4). While eight-cell-stage blastomeres are more likely to form EPI, the more mature 16-cell-stage blastomeres that have spent more time on the outside of the embryo are biased towards PE (figure 4*d*).

**Figure 5.**
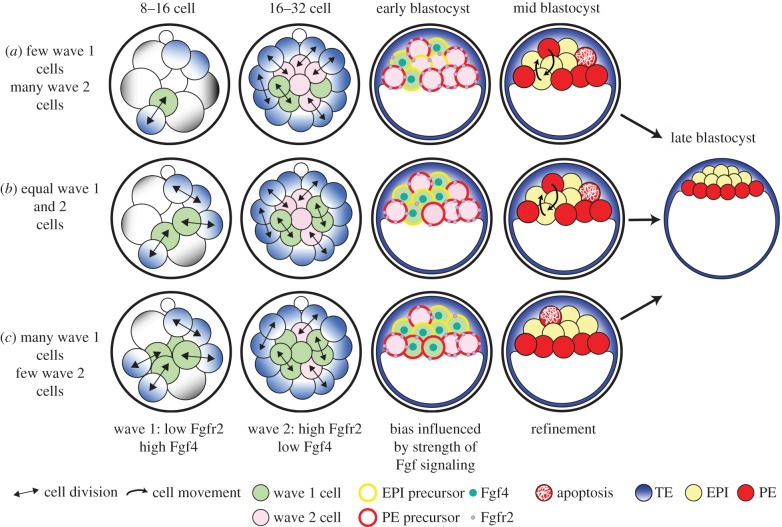
ICM cell fate is influenced by time of internalization and the local Fgf signalling context. Model for ICM lineage specification. Wave 1 inside cells express Fgf4, whereas wave 2 inside cells express higher levels of Fgfr2. (*a*) In embryos where there are few wave 1 inside cells, the ICM is predominantly composed of wave 2-generated cells, and therefore there is little Fgf4. All wave 1-derived cells give rise to EPI and owing to the low levels of Fgf signalling a few wave 2-generated cells also form EPI, although the majority form PE. (*b*) In embryos where there are equal numbers of wave 1 and 2-generated cells, and therefore a balance of Fgf4 and Fgfr2-expressing cells in the ICM, a developmental bias based on internalization time is most apparent. (*c*) In embryos where there are many wave 1 inside cells, the ICM is predominantly composed of these cells and the levels of Fgf4 are correspondingly high. All wave 2-generated cells form PE, as well as some wave 1 cells.

Overall our results provide a potential mechanistic model for the specification of PE and EPI precursors in the mouse ICM (figure 5). Inside cells generated in the first wave of asymmetric divisions express Fgf4, whereas those generated by the second wave of asymmetric divisions express Fgfr2, making them more susceptible to Fgf4 signalling than wave 1 cells, and therefore biased towards the PE lineage. The strength of this bias on final lineage contribution by cells generated in each wave is tempered by the specific ICM composition of each embryo. Our results indicate that most frequently there is an approximately equal balance of wave 1- and 2-derived cells in the ICM, Fgf signal producers and responders respectively, and in this case we observe a clear developmental bias (figure 5*b*). In those embryos with few wave 1-derived cells (less than 30%), there will be less Fgf4 in the ICM, and therefore the impact of Fgf4 signalling on the cells with high Fgfr2 expression may not be as strong and some will form EPI (figure 5*a*). In embryos with many wave 1-derived cells (more than 70%) there will be high levels of Fgf4 in the ICM, driving the cells with high Fgfr2 expression, as well as some with lower expression, towards the PE lineage (figure 5*c*). It is important to note that this initial allocation of PE and EPI precursors is by no means binding, the cells are not yet ‘committed’ and are capable of forming either lineage if given the right cue, in an agreement with the regulative nature of preimplantation development and with earlier work [3]. It is possible, however, that as the inside cells generated in the second wave of asymmetric divisions are the daughters of more mature outside cells that have begun to differentiate into TE, they may be comparatively less pluripotent than wave 1 inside cells, which is reflected in their bias towards forming the more differentiated ICM lineage, the PE. Here, we show that wave 1 and 2 inside cells are inherently different and provide a model for ICM lineage specification that combines the effect of biases owing to internalization time, with the unique ICM environment of each embryo, and the regulative capability of early mouse development.

## 5. Material and methods

### 5.1. Embryo culture and inhibitor treatment

Mouse embryos were collected from four- to six-week-old superovulated F1 (C57B16 × CBA) females mated with F1 males and cultured in KSOM medium in 5% CO_2_ as previously described [3]. For Fgf signalling inhibition experiments, eight-cell embryos were cultured in KSOM containing PD173074 (100 nM, Cayman Chemical). Control embryos were cultured in KSOM containing an equivalent volume of DMSO.

### 5.2. Immunostaining and fluorescent *in situ* hybridization

Immunostaining and FISH were performed as described previously [22]. Primary antibodies used were goat anti-Sox17 (R&D Systems), rabbit anti-Fgfr2 (Santa Cruz) and rabbit anti-Nanog (2B Scientific). To identify inside cells generated by different waves of asymmetric cell divisions, individual blastomeres of eight-cell stage embryos were injected with *EGFP* mRNA(400 ng μl^−1^) and monitored to determine division orientations before being fixed for immunostaining at the early blastocyst stage. Images were taken using Zeiss LSM5100 or Leica SP5 confocal microscopes, and all image processing, intensity measurements and cell counting were performed using ImageJ (http://rsbweb.nih.gov/ij/).

### 5.3. Overexpression of Fgfr2

To overexpress Fgfr2, full-length ORF *Fgfr2* (transcript variant IIIc) was cloned into pRN3P as previously described [23]. One blastomere of two-cell stage embryos was injected with *Fgfr2* mRNA (100 ng μl^−1^) and *EGFP* mRNA (400 ng μl^−1^) or *Tomato* mRNA (400 ng μl^−1^) as lineage tracers or in controls with tracer mRNA alone. Successful overexpression of Fgfr2 was confirmed by immunostaining.

### 5.4. Generation of chimaeric embryos

To make chimaeras containing one labelled eight-cell, or 16-cell outside blastomere in the inside of the embryo, superovulation injections and matings were staggered by 12 h, under reverse-light conditions so that eight-cell-stage and 16-cell-stage embryos could be manipulated at the same time. Embryos were recovered at the two-cell stage and those for the inside ‘donor’ cells were injected with *Tomato-RFP* mRNA (400 ng μl^−1^) or *EGFP* mRNA (400 ng μl^−1^) into both blastomeres. To make chimaeras with an eight-cell-stage blastomere inside, the zona pellucida was removed from fluorescently expressing eight-cell embryos and unlabelled ‘host’ eight-cell-stage embryos by Acid Tyrode's treatment and the embryos disaggregated in cation-free M2 by gentle manipulation with a narrow glass pipette. The blastomeres were then incubated in phytohaemagglutinin (150 μg ml^−1^ in BSA-free M2) for 10 min and the donor cells surrounded by host cells. For each chimaera, we used up to 16 eight-cell host blastomeres to ensure that the donor cell was completely enclosed inside the embryo. The chimaeras with 16-cell-stage outside blastomeres positioned inside were made in the same way but the embryos were incubated in a fluorescently labelled 0.2 μm microsphere suspension (Polysciences, Inc.) [4] diluted to 1 : 50 for 30 s prior to disaggregation in order to label outside cells. When the embryos were disaggregated, the outside (fluorescently labelled) cells could be selected for chimaera generation. The chimaeras were cultured in KSOM until E4.5, fixed in 4% paraformaldehyde and the contribution of the fluorescent ‘donor’ cells to each ICM lineage assessed by position and immunostaining for Sox17 and Nanog.

### 5.5. Statistical analysis

Cell numbers are visualized as average number with standard deviation. Significance was calculated using two-tailed Student's *t*-test.

## Supplementary Material

Supplementary Figures
