# Supplementary material for: The differential response to Fgf signalling in cells internalized at different times influences lineage segregation in preimplantation mouse embryos
Source: Open Biol. 2013 Nov;3(11):130104. doi: 10.1098/rsob.130104 (PMC3843820; doi:10.1098/rsob.130104)
Supplement: Supplementary Figures [file rsob130104supp1.pdf]

Supplementary Figures

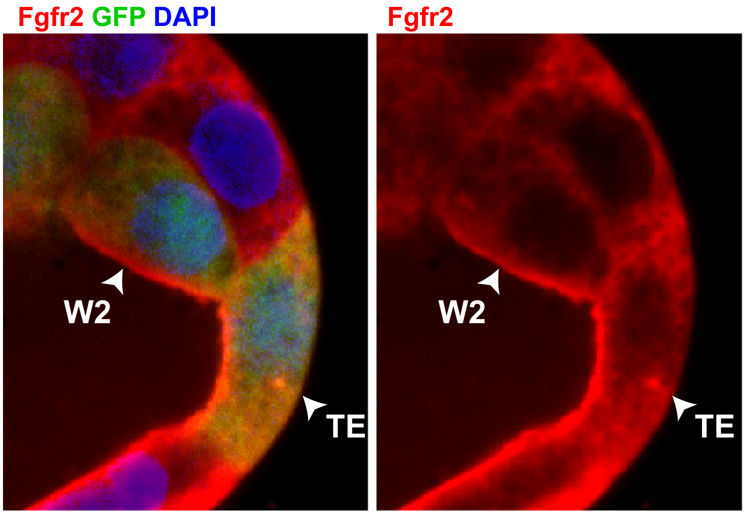

Figure S1. Expression of Fgfr2 in a wave 2 derived inside cell positioned at the surface of the ICM. Detail of the adjacent TE cell shows that Fgfr2 expression detected in the wave 2 inside cell is not associated with trophectodermal processes belonging to the adjacent TE cell.

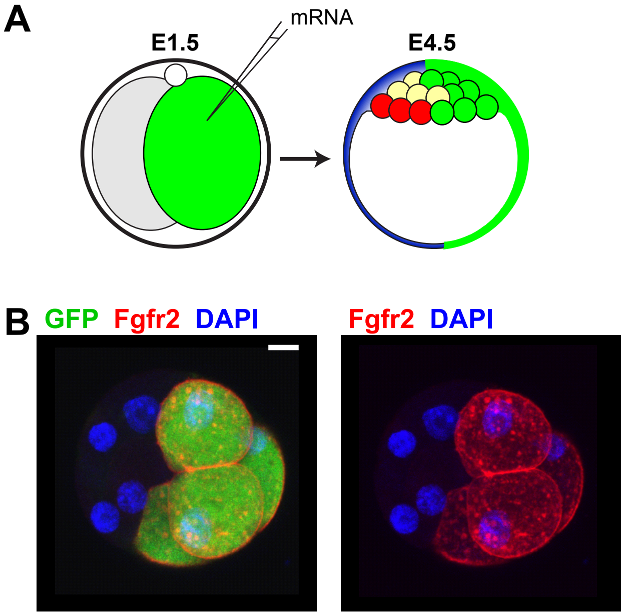

Figure S2. (A) Overexpression in half of the embryo by injection of mRNA to one blastomere at the 2-cell stage. (B) Confirmation of Fgfr2 overexpression at the 8-cell stage. Injected cells are marked by GFP expression.

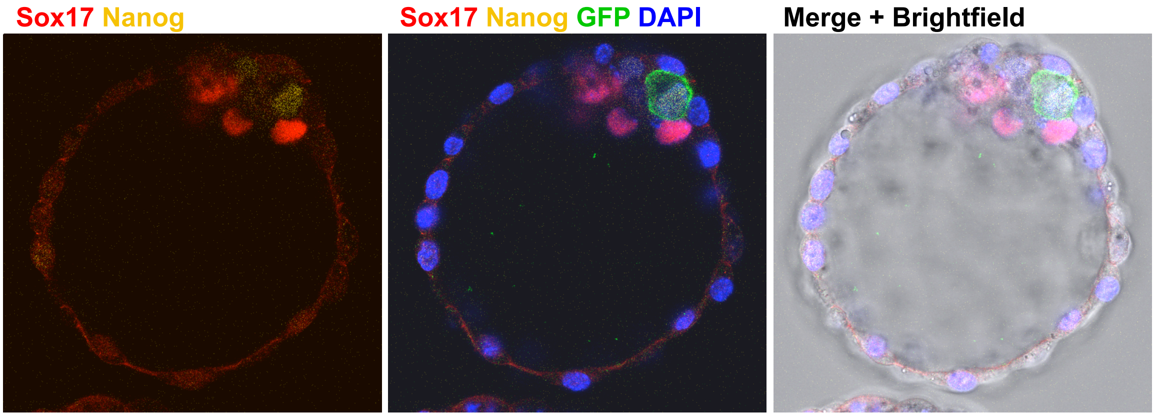

Figure S3. Example of a chimera made with an 8-cell blastomere placed on the inside of the embryo, fixed at E4.5 and immunostained for Sox17 (PE) and Nanog (EPI). GFP expression marks progeny of the donor cell.
